# Noncovalent Interactions in the Catechol Dimer

**DOI:** 10.3390/biomimetics2030018

**Published:** 2017-09-13

**Authors:** Vincenzo Barone, Ivo Cacelli, Alessandro Ferretti, Giacomo Prampolini

**Affiliations:** 1 Scuola Normale Superiore di Pisa, Piazza dei Cavalieri, I-56126 Pisa, Italy; 2 Dipartimento di Chimica e Chimica Industriale, Università di Pisa, Via G. Moruzzi 13, I-56124 Pisa, Italy; ivo.cacelli@unipi.it; 3 Istituto di Chimica dei Composti OrganoMetallici (ICCOM-CNR), Area della Ricerca, Via G. Moruzzi 1, I-56124 Pisa, Italy; ferretti@iccom.cnr.it (A.F.); giacomo.prampolini@pi.iccom.cnr.it (G.P.)

**Keywords:** noncovalent interactions, catechol, aromatic dimers, computation, electronic correlation, dispersion

## Abstract

Noncovalent interactions play a significant role in a wide variety of biological processes and bio-inspired species. It is, therefore, important to have at hand suitable computational methods for their investigation. In this paper, we report on the contribution of dispersion and hydrogen bonds in both stacked and T-shaped catechol dimers, with the aim of delineating the respective role of these classes of interactions in determining the most stable structure. By using second-order Møller–Plesset (MP2) calculations with a small basis set, specifically optimized for these species, we have explored a number of significant sections of the interaction potential energy surface and found the most stable structures for the dimer, in good agreement with the highly accurate, but computationally more expensive coupled cluster single and double excitation and the perturbative triples (CCSD(T))/CBS) method.

## 1. Introduction

Nowadays, there is a general consensus about the primary role played by noncovalent interactions, in particular those involving aromatic rings, in molecular, life, and materials sciences. In addition to being responsible for key biological processes that range from base stacking in deoxyribonucleic acid (DNA) [1], to the color of red wine [2] and, more generally, food quality [3], it is of the foremost importance to understand, rationalize and, hence, exploit their features in cutting-edge applications as advanced catalysis [4,5], biomedical materials [6,7] and novel drugs design [8], advanced organic photovoltaics [9,10,11,12,13], complex self-assembled structures [14], or bio-nano-materials [15,16]. Such ubiquity of the aromatic interactions has often inspired multidisciplinary research [17], aimed to exploit their peculiar features in the design and construction of biomimetic materials. From a physical point of view, noncovalent interactions among molecules bearing aromatic moieties originate from a variety of different forces, including π-stacking, XH–π or charge-transfer (CT), besides the ubiquitous dispersion. Furthermore, the presence of additional functional groups can introduce other kinds of interactions (like e.g., hydrogen (HB) or halogen bonds), leading to nontrivial interference effects, which tune both the structure and the properties of the resulting material. In this framework, computational methods can play a crucial role for rational design and interpretation, provided that they are able to couple reliability, feasibility, and ability to unravel the different contributions [18,19]. It should be also mentioned that, although the embedding environment is often neglected, or only roughly approximated in most computational studies, its effect can be significant or even decisive in biomimetic processes. However, comprehensive studies of pairs of interacting species in the gas phase are a mandatory starting point for unraveling the weight of the different effects.

In the past few years, catechol has attracted increasing attention as a precursor of bio-inspired materials [20,21,22,23,24,25,26]. From a theoretical point of view, catechol is an ideal candidate to test the capability of new computational approaches to accurately represent the delicate balance among the different kinds of noncovalent interactions, occurring in the presence of catechol units. In fact, apart from the π-stacking and XH–π interactions due to the aromatic core, interactions between these species are also characterized by the insurgence of strong (OH–H) and weak (OH–π) HB patterns, which may play an important role in the supramolecular assembling. The main problem is that aromatic interactions are dominated by dispersion forces that standard electronic calculations have difficulty to reproduce. Indeed, in the past ten years, much effort has been devoted to the development of approaches that overcome the problem [27,28,29,30,31,32,33,34,35,36,37,38,39,40,41,42,43]. Within the framework of density functional theory (DFT), attempts have been made to set appropriate functionals which incorporate the effects of dispersion, such as that of Truhlar et al. [43] or to introduce semi-empirical atomistic corrections, as suggested by Grimme and coworkers [30,32,33]. Among wave function (WF)-based approaches, the most accurate but also computationally most expensive method is the coupled cluster approach including a full account of single and double excitations together with perturbative inclusion of connected triple excitations, and extrapolation to the complete basis set limit (CCSD(T)/CBS) [4,19,34,37,38,41,44,45,46,47,48,49,50]. Still within a WF framework, perturbative second-order Møller–Plesset (MP2) calculations could be carried out at a much lower computational cost, yet it is well known [40] that they tend to overestimate aromatic binding energies, especially when employed with large basis sets. These inaccuracies can be overcome by resorting to an idea proposed almost forty years ago by Kroon-Batenburg and Van Duijneveldt [51] and successively refined by Hobza and Zahradnik [52], based on the use MP2 calculations with the small 6-31G* basis set, modified by reducing to 0.25 the exponent of the *d* polarization function placed on each carbon atom of the benzene dimer. Such an approach, often referred to as MP2/6-31G*(0.25), was then fully validated with reference to interaction energies of benzene and a few other aromatic dimers computed at the CCSD(T)/CBS level [53,54,55,56,57,58,59,60,61]. More recently, the method has been generalized to different basis sets, and applied to several molecular prototypes, including liquid crystals [62,63], pyridine [64], quinhydrone [27], dihydroxyindole derivatives relevant in eumelanin formation [65], and, very recently, to small aromatic heterocycles [66], where the procedure to find the suitable modified basis sets, labeled MP2^mod^, has been automated and extended to the optimization of the orbital exponents of *d* functions on heteroatoms and *p* functions on hydrogen, within the 6-31G** basis set.

Here, the MP2^mod^ method is applied to the catechol dimer in the gas phase. First, MP2^mod^ accuracy is validated against high-quality CCSD(T)/CBS predictions, purposely carried out for a number of selected geometries of catechol dimers. Next, MP2^mod^ is employed in the exploration of the catechol’s interaction potential energy surface (IPES), with the aim of finding the optimal structure of the dimer by a comparison of different possible arrangements. This allows us to investigate the different roles played by HB and π-stacking interactions in the dimer formation. Incidentally, it might also be of interest, following Wheeler group’s suggestions [4,44,45], to verify if noncovalent interactions in catechol can be correlated to the simple direct interaction between the (hydroxyl) substituents, or if, on the contrary, a rationalization of the resulting interaction patterns requires a more complex analysis, taking into account the specific role of each contribution. 

The catechol dimer has also been studied at the DFT level by Estévez et al. [67], who considered structures determined either by X-ray measurements or by geometry optimizations at the MPW1B95/6-311++G(2d,2p) level. In the following these results will also be discussed in comparison with our findings.

## 2. Computational Details

The full geometry optimization of the catechol monomer has been performed by DFT, at the B3LYP/*aug*-cc-pvDZ level, by minimizing the energy with respect to all internal coordinates. Unless otherwise stated, the internal monomer’s geometry was kept unaltered in all subsequent calculations.

As far as the intermolecular energy is concerned, reference CCSD(T)/CBS calculations have been carried out on catechol dimers following the protocol adopted in previous works [27,36,66], which can be summarized as follows:

1. The difference Δ*_CC-MP2_* between CCSD(T) and MP2 interaction energy is evaluated using for both calculations the Dunning’s correlated *aug*-cc-pvDz basis sets:
  (1) $$\Delta_{CC-MP2}={\left|\Delta E_{}^{CCSD\left(T\right)}\right|}_{aug-cc-pvDz}-{\left|\Delta E_{}^{MP2}\right|}_{aug-cc-pvDz}$$
2. The MP2 energy in the CBS limit, $\Delta E_{CBS}^{MP2}$, is computed through the extrapolation scheme proposed by Halkier et al. [68], making use of the *aug*-cc-pvDz and *aug*-cc-pvTz basis sets. Despite the state-of-the-art extrapolation procedure [37,41,50] is often carried out with the larger *aug*-cc-pvTz and *aug*-cc-pvQz basis sets, it has been recently shown that, for similar aromatic dimers, the use of the smaller *aug*-cc-pvDz and *aug*-cc-pvTz affects the computed interaction energies by few hundredths of kcal/mol [66]. In consideration of the fairly large number of dimers investigated and the computational cost of a CCSD(T) calculation at the *aug*-cc-pvQz level, the smaller sets (Dz and Tz) were chosen as the best compromise between accuracy and feasibility.
3. Finally, the CCSD(T)/CBS interaction energy, $\Delta E_{CBS}^{CCSD\left(T\right)}$, is recovered as:
  (2) $$\Delta E_{CBS}^{CCSD\left(T\right)}=\;\Delta E_{CBS}^{MP2}+\;\Delta_{CC-MP2}$$
4. All energies were corrected for the basis set superposition error (BSSE) with the standard counterpoise (CP) correction [69].

The MP2^mod^ exponent optimization was performed by means of the Exopt code [27,36,66], by minimizing the objective function I:

(3) $$I\left(\bar{P}\right)=\;\frac{1}{N_{geom}}\overset{N_{geom}}{\underset{k=1}{\sum }}{\left[\Delta E_{CBS}^{CCSD\left(T\right)}-\Delta E_{}^{MP2^{mod}}\left(\bar{P}\right)\right]}^{2}$$

where *N_geom_* is the number of considered dimer geometries and $\bar{P}$ the vector containing the basis sets exponents to be optimized. All the MP2^mod^ calculations were carried out with the 6-31G** basis set, and the exponents of the *d* functions on heavy atoms and the *p* functions on H were optimized. Further details on the optimization protocol can be found in [66] and are also briefly commented in the next section. In all MP2^mod^ calculations, the CP correction was applied to take care of the basis set superposition error.

Finally, to better compare with the results reported by Estévez et al. [67], the interaction energy of selected dimer arrangements was also computed at the DFT level, using the same procedure employed in [67]: the MPW1B95 functional was employed, together with the 6-311++G(2*d*,2*p*), while no correction was applied to take care of the BSSE. 

All CCSD(T), MP2, MP2^mod^ and DFT calculations were carried out with the Gaussian09 software package [70].

## 3. Results and Discussion

### 3.1. MP2^mod^ Tuning and Validation

**Figure 1:**
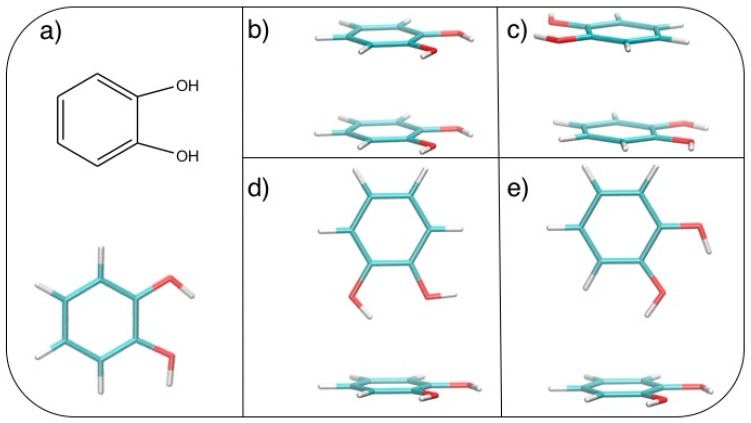
(**a**) Catechol structural formula (**top**) and graphical representation (**bottom**). Stacked dimers: (**b**) face-to-face (FF) and (**c**) antiparallel face-to-face (AFF); T-shaped (TS) dimers: (**d**) TS_1_ and (**e**) TS_2_. C: Cyan; H: White; O: Red.

After geometry optimization, the catechol monomer is planar with the two hydroxyl hydrogens pointing in the same direction (see Figure 1a). 

**Figure 2:**
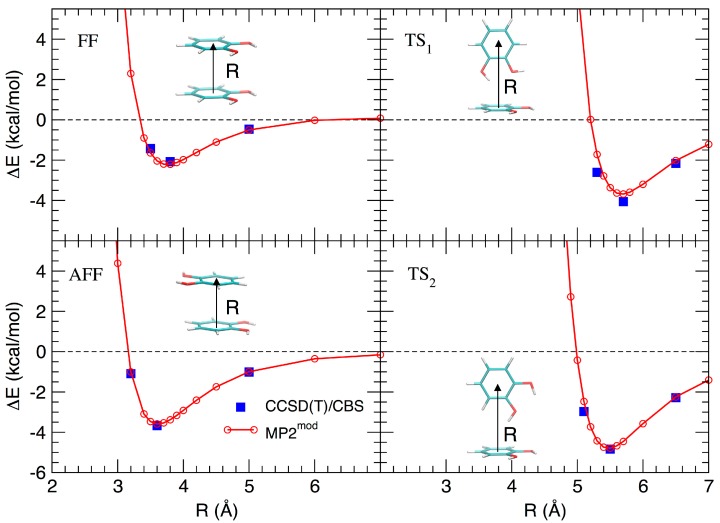
Comparison between the ‘best exponent’ and CCSD(T)/CBS for the interaction energy profiles obtained by displacement of the four structures shown in Figure 1.

Based on the results recently achieved for several heteroaromatic dimers, where stacked and T-shaped (TS) conformers where found to be the most stable, four starting arrangements have been set up by placing the two monomers at different distances and relative orientation. Namely, the face-to-face (FF, Figure 1b), the antiparallel face-to-face (AFF, Figure 1c), and two TS conformations, one with both hydroxyls (TS_1_, Figure 1d) and one with only one hydroxyl (TS_2_, Figure 1e) pointing towards the other ring. Following the protocol recently developed in our group [66], the MP2^m^^od^ best exponents were determined as follows: starting from each of the four selected conformations, a set of dimer arrangements was created by displacing one monomer along a selected coordinate R, defined as the line connecting the centers of the two rings, as shown in the insets of Figure 2. Next, an estimate (data not shown) of the interaction energy (ΔE) of the resulting dimer geometries was obtained at the MP2^m^^od^ level, employing the basis set recently optimized by us for quinhydrone [27], thus obtaining preliminary interaction energy profiles. Three points (displayed as blue squares in Figure 2) were selected for each profile (namely one in the minimum, one in the short distance range and one in the attractive branch of the curve) and the corresponding CCSD(T)/CBS interaction energies were computed and used to build a reference database containing 12 elements. This database was then used for the optimization of the exponents of the polarization functions of the 6-31G** basis sets suitable for MP2^mod^ calculations. The starting exponents of the standard 6-31G** basis set are 0.80 for *d* functions on carbon and oxygen and 1.1 for *p* functions on hydrogen. After optimization, the best exponents were found to be 0.27 and 0.34 for the *d* functions on carbon and oxygen, respectively, and 0.36 for *p* functions on hydrogen. The final standard deviation, $\sqrt{I}$, see Equation (3), resulted to be less than 0.3 kcal/mol with respect to the CCSD(T)/CBS energies. 

The resulting MP2^mod^ curves are displayed in Figure 2, together with the reference values. The excellent agreement between the two methods, in line with the results previously obtained for similar molecules, allows us to apply rather confidently the MP2^mod^ method to the study of the catechol dimer. According to both CCSD(T)/CBS and MP2^mod^ results, the most stable structure is the TS_2_ one (around −5.0 kcal/mol), with the minimum at a slightly smaller value of R (5.4 Å), with respect to the similar TS_1_ conformer (5.6 Å), which is in turn almost as stable (≈−4.0 kcal/mol) as the antiparallel stacked conformer (AFF, −3.8 kcal/mol). Among the two stacked conformations, FF and AFF, the second one is more stable, in agreement with the repulsive interaction between the OH dipoles in the FF form. 

### 3.2. Stacked Cathecol Dimers

**Figure 3:**
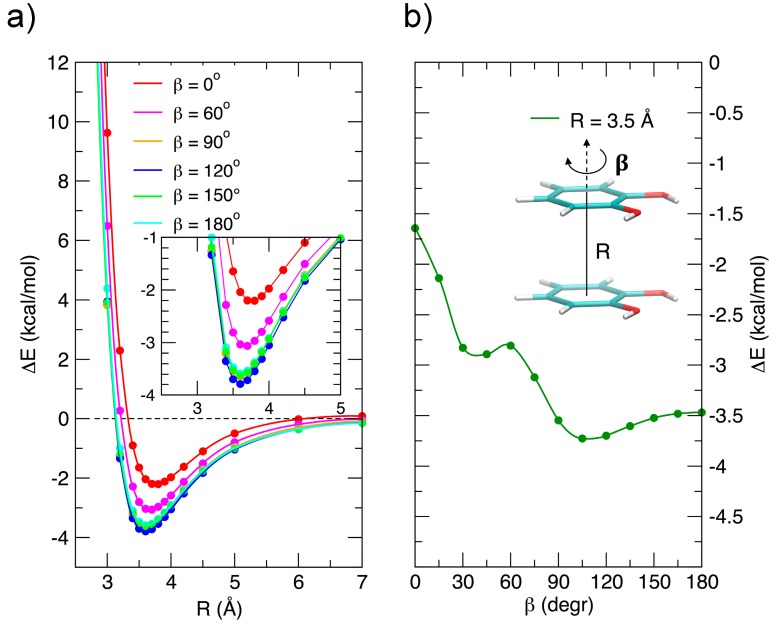
MP2^mod^ results for the stacked configurations. (**a**) Interaction energy as a function of the inter-ring separation *R* for different *β* angles. (**b**) Interaction energy as a function of the *β* angle at the ring–ring separation (*R* = 3.5 Å) corresponding to the minimum energy.

Due to its importance, the stacked arrangement has been studied with some care as a function of the ring–ring distance *R* and of the angle *β*, which expresses, as shown in Figure 3b, the relative rotation of the two rings with respect to the line connecting their centers. The relevant results are reported in Figure 3. In the left panel, the interaction energy, reported vs. *R* for assigned rotation angles, shows minima at similar *R* values for all angles, and a marked dependence on *β* at low vales (from 0 to 60°), whereas for *β* > 90° the curves are close to each other: at the minimum the interaction energy changes by only ≈0.25 kcal/mol in the range 90–180°. Although this behavior seems roughly consistent with a dipole–dipole interaction, the resemblance of the 90, 120, 150, and 180° curves is an indication that higher multipoles, or, equivalently, local dipoles, should play a role in an electrostatic rationalization of the observed energy curves. This is in agreement with the idea of Wheeler and coworkers [45,46] that stacking interaction in substituted aromatic species is strongly influenced by the local interaction of the substituents, rather than to changes induced in the π electronic density upon substitution, as suggested by older models.

Figure 3b shows the energy variation as a function of *β* and connects the FF (*β* = 0°) to the AFF (*β* = 180°) arrangement at a fixed ring-ring distance (*R* = 3.5 Å). The curve shows a not monotonic behavior, probably due to the presence of two functional groups, with an absolute minimum near 110°, rather than at 180°, as could be expected for single substituted benzene rings. However, despite the perturbations triggered by the specific interaction among the two strong local dipoles of the monomers, the transition from FF to AFF arrangements along *β* is rather marked and clearly indicates a preference for antiparallel stacked arrangements, as already put in evidence in Figure 2.

**Figure 4:**
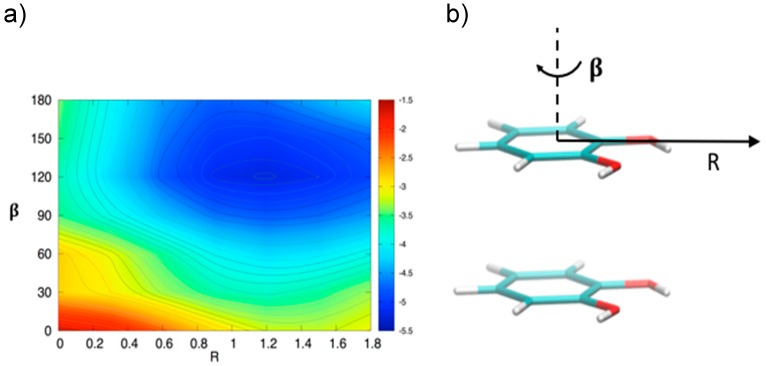
(**a**) Two-dimensional scan of the catechol interaction potential energy surface (IPES) in stacked conformations, performed at the MP2^mod^ level. (**b**) The IPES section was sampled by varying the angle *β*; the displacement *R* is also shown.

In order to gain a deeper insight into the orientation dependence of the stacking forces in the catechol dimer, taking advantage from the low computational cost of the MP2^mod^ method, we can explore different sections of the catechol IPES. For instance, in Figure 4 a two-dimensional contour plot of the interaction energy (Δ*E*) is reported as a function of the horizontal displacement of the two rings (*R*) and of the rotation angle (*β*) of one of the two rings around the perpendicular axis, at the inter-ring distance of 3.5 Å (i.e., the position of the minimum for the stacked energy curves reported in Figure 3). 

Figure 4 clearly shows that the dimer is much more stable when displaced and rotated with respect to the FF arrangement, with a minimum at *R* ≈ 1.2 Å and *β* ≈ 130°. It is noteworthy that the effects of horizontal displacement (i.e., varying *R*) and *β* rotation can be ascribed to different origins, closely related to the catechol molecular structure. In fact, the increase of the binding energy upon displacement closely resembles the well-known behavior of the benzene dimer [47,49,50] originated from a “pure” aromatic interaction: shifting one monomer along the *R* coordinate diminishes the quadrupolar repulsion between the two rings [49], whereas the attractive dispersion interaction decreases to a lesser extent, hence resulting in a global increase of the binding energy [47,49]. As discussed above, the energy profile vs. *β* rotation is strictly connected with the presence of OH substituents, as suggested by the net increase of the interaction energy in going from a parallel (*β* = 0°) to an antiparallel (*β* = 180°) arrangement.

**Figure 5:**
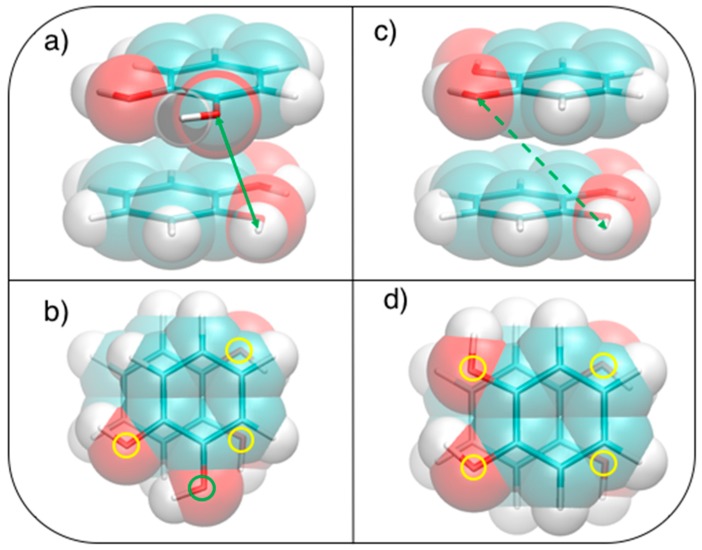
Stacked displaced (*R* = 1.2 Å) geometries at (**a**,**b**) *β* = 120° and (**c**,**d**) *β* = 180°. (**a**,**c**): Side view, H–O distances of (**a**) 3.7 and (**c**) 5.0 Å are indicated with a green arrow ; (**b**,**d**): Top view, the position of oxygen atoms is shown with colored circles, distinguishing more (yellow) or less (green) interacting ones.

This simple picture is consistent with the minimum of −5.2 kcal/mol (*R* = 1.2 Å, *β* = 120°) in a displaced near antiparallel configuration, not coincident with the perfect antiparallel arrangement (*β* = 180°) where the MP2^mod^ interaction energy is −4.7 kcal/mol. This subtle difference can find a rationale at a closer look of the molecular structure, embracing Wheeler’s idea that unexpected substituents effects can be explained by considering their direct interaction with the neighboring cloud of the other ring [44,45,46]. The *β* = 120° and *β* = 180° conformers are displayed in Figure 5. In Figure 5b,d, where a top view of both dimers is shown, the positions of the oxygen atoms are marked with colored circles, to put in evidence the differences between the two arrangements. It appears as in the *β* = 180° geometry all oxygen atoms lie approximately above a C=C bond of the other ring, resulting in an unfavorable electrostatic interaction with the carbon *π* orbitals, while at *β* = 120° only three oxygen atoms contribute to such repulsive term. Consistently, the Hartree–Fock contribution to the total MP2^mod^ energy, which is repulsive in both cases, increases by 1 kcal/mol, in going from *β* = 120° (3.3 kcal/mol) to *β* = 180° (4.3 kcal/mol). Finally, another possible source of attractive interaction comes from the HB interaction between the hydrogen atom of one hydroxyl group and the closest oxygen of the other ring, as evidenced in Figure 5a,c, where it appears as in the *β* = 120° conformer the hydrogen atoms lie at much closer distances (3.7 Å).

### 3.3. T-Shaped Cathecol Dimers

As shown in Figure 2, another kind of arrangement which can compete with the stacked geometries discussed above is the TS configuration. In this case, most of the interaction energy is expected to come from XH–π forces, in particular when two or one hydroxyl groups point towards the other ring’s plane, as in the TS_1_ and TS_2_ geometries. 

**Figure 6:**
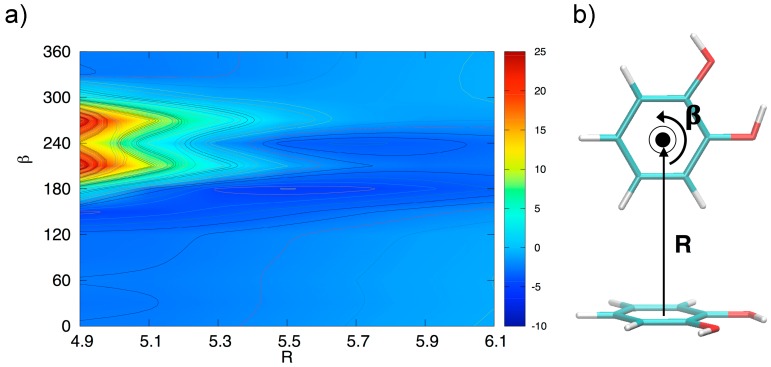
(**a**) Two-dimensional scan of the catechol IPES in TS conformation, performed at MP2^mod^ level. The IPES section was sampled by varying the angle *β* and the displacement *R* shown in (**b**), where the TS arrangement at *β* = 0° is displayed. The *β* rotation is performed as indicated by the black arrow (e.g., for *β* = 240° the dimer is found in the TS_1_ geometry shown in the right top panel of Figure 2).

In order to verify this assumption, the MP2^mod^ computational feasibility has been exploited once again to explore an additional IPES section, related to the TS conformers and shown in Figure 6. At small inter-ring distances, the dependence on *β*-rotation is striking and the most favorite conformer at *R* = 4.9 Å is found at *β* = 0° (i.e., the TS arrangement shown in Figure 6b), with the interaction energy (−2.4 kcal/mol) very similar to the value reported for the benzene dimer in the same configuration [49,50,53,57,71]. Conversely, due to the small distance between the H hydroxyl atom and the other catechol ring (see for instance TS_1_ in Figure 2), the interaction energy in the 180–300° range is repulsive, with a maximum of almost 25 kcal/mol at *β* = 270°. The situation changes dramatically by increasing *R*, as in the 180–300° range the interaction energy shows a much steeper gradient. In fact, the IPES section minimum is found in a TS conformation at *β* = 270° and *R* = 5.5 Å, where the hydroxyl group points towards the other ring plane similarly to the TS_2_ arrangement shown in the right bottom panel of Figure 2, resulting in a total interaction energy of −5.1 kcal/mol.

### 3.4. Effect of the Hydrogen Bond

**Figure 7:**
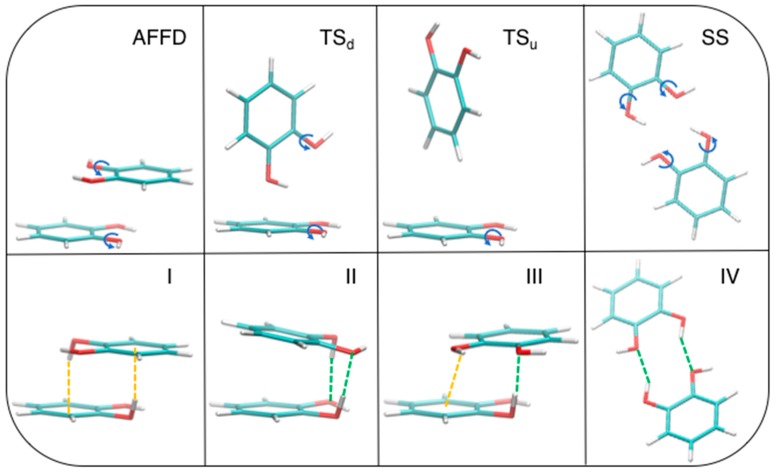
MP2^mod^ geometry optimization starting from the displaced AFF (AFFD), TS_d_, and TS_u_ conformations (top panels). The corresponding optimized structures, I, II, III, and IV, are displayed in the bottom row. The rotated hydroxyl groups are evidenced in the top panel with a blue arrow, while the atoms involved in OH–O and OH–π interactions are connected in the bottom panels by green and orange dashed lines, respectively.

The above described competition between stacked and TS geometries misses although another player, which could significantly alter the delicate balance between them. In fact, apart for a small contribution to the stability of the *β* = 120° conformer in the stacked conformations, the HB contribution was never decisive to the total interaction, due to too large distances between the involved hydrogen and oxygen atoms, which could be reduced by allowing internal rotation around C–O bonds. In order to find even more stable structures, we have released such constraint and performed a full optimization at MP2^mod^ level, starting from four different conformations (see Figure 7, top panels). The first starting geometry is a displaced AFF (AFFD). Next, two TS structures were prepared, with one or both hydroxyl groups pointing down towards the other ring (TS_d_ and TS_u_, respectively). Notice that the latter is very similar to that taken from crystallographic data and investigated by Estévez et al. [67]. Finally, a fourth arrangement was built from scratch, where the two rings are placed in side-by-side (SS) conformation, with both hydroxyl groups resulting at close distance, thus maximizing the effect of HBs. All optimizations ended up successfully in four different local minima, as confirmed by a frequency calculation purposely carried out for each of the resulting structures. The corresponding optimized structures are shown in the bottom panel of Figure 7 as I, II, III, and IV, respectively.

Dimer formation does not result in large changes in the internal geometry of each catechol monomer. Bond lengths within each monomer change by less than 0.03 Å and the backbone remains planar. For each ring, only one hydroxyl hydrogen moves out of plane, establishing OH–O or OH–π interactions, while the other O–H bond remains nearly coplanar with the ring, due to the formation of an intramolecular OH–O HB with the closest oxygen atom (in Figure 7, geometry II it is out of plane by only 13°). The dihedral angle which drives the position of the out of plane hydrogen is 66° for I, 71.5° for II, and 68.7° for III, whereas the other ring hydrogen does not rotate. The final conformations reveal that the internal rotation has a significant effect on the interplay of the different interaction terms. In fact, as evident from Figure 7, both TS conformations are not stable upon a full optimization, and eventually end up in a stacked arrangement, whereas the AFFD conformer undergoes to the expected rotation from *β* = 180° to *β* ≈ 120°, but maintains the stacking arrangements. The OH–O interaction plays the major role in TS_d_, which becomes II, while OH–π weak HBs guide the hydroxyl rotation and are prevalent in AFFD, which becomes I. Although less stable, the last optimized conformer III, is characterized by a single hydroxyl rotation, which allows the insurgence of a HB (green dashed line in Figure 7), while the other hydrogen remains coplanar to the ring, yet interacting with the other monomer establishing a OH–π noncovalent bond. For the conformation IV, geometry optimization results in a structure which is again less stable than I and II and somewhat more stable than III (see Table 1). In this case, the hydroxyl hydrogens undergo a small rotation with respect to the initial conformation and the two rings are slightly displaced out of the plane that initially (see SS) contained both monomers. 

The interaction energies for the four final structures are reported in Table 1, along with the value computed at the same geometries with the MPW1B95/6-311++G(2*d*,2*p*) in [67], as well as with the “gold standard” CCSD(T)/CBS. From these data, it is clear that the most stable structure is II, which differs by only 1.6 kcal/mol from I, whereas III and IV are far higher in energy. The agreement between the MP2^mod^ values and their CCSD(T)/CBS counterparts is very good, especially considering that these latter geometries are outside the MP2^mod^ training set, while the computational advantage of using MP2^mod^ with small basis sets is apparent from the last three columns. Surprisingly, the MPW1B95 functional severely underestimates the reference CCSD(T)/CBS interaction energies, yielding, in the present case, only a qualitative correct description, at least according to the protocol provided in [67]. 

**Figure 8:**
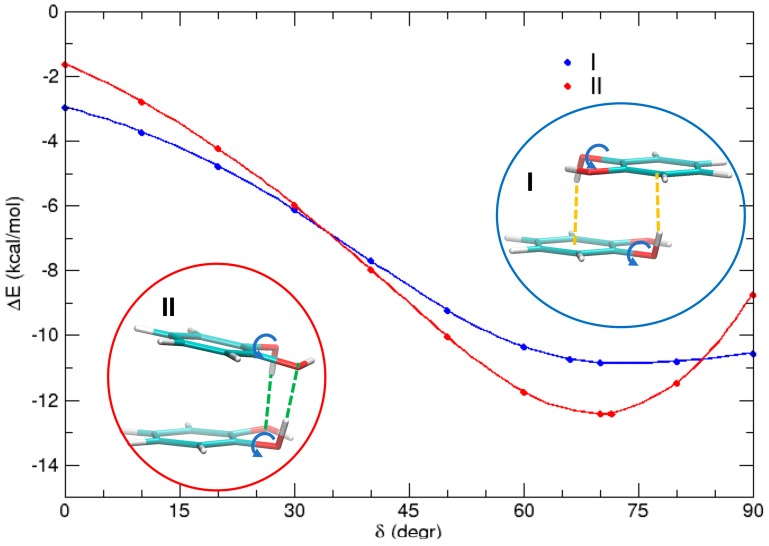
MP2^mod^ scans of the HOCC dihedral (*δ*) for conformers I (blue) and II (red), highlighting the role of OH–O and OH–π interactions, indicated with orange and green dashed lines, respectively.

Finally, it is interesting to investigate the different HB contributions in the two most stable conformations I and II. This can be done by performing a rigid scan of the rotation angle *δ* of the two hydrogen atoms with respect to the C–O bond in both conformations (*δ*). The results are shown in Figure 8. For *δ* = 0° (i.e., when each hydrogen is coplanar to the aromatic ring), dispersion interactions are the main source of attraction, although perturbed by the electrostatic interaction between the dipoles, which favors dimer I (in an antiparallel alignment) by ≈1 kcal/mol with respect to dimer II. As *δ* increases, both the hydrogen atoms involved in the rotation come to closer distances from the other monomer, and may establish HBs. These noncovalent interactions remarkably stabilize both complexes, by almost 7 kcal/mol in I and more than 10 kcal/mol in II. These differences can find a rationale by looking at the insets of Figure 8. In dimer I, since each hydrogen points approximately towards the center of the neighboring ring, two weak HBs of the OH–π type are settled whereas, in dimer II, both hydrogen atoms are involved in a stronger OH–O HB. As a consequence, the minimum of the latter conformer is stabilized by ≈2 kcal/mol with respect to I.

## 4. Conclusions

In this paper, we have reported our study of the intermolecular landscape of a catechol dimer with a two-fold interest. On the one hand, noncovalent interactions, and especially those involving aromatic rings, govern many biological processes and it is, therefore, of basic importance to reach a good comprehension of the different role that the various forces play in specific systems. On the other hand, noncovalent interactions are still a challenging benchmark for standard computational methods, hence, it can be significant to exploit dedicated approaches. 

Catechol is well known to be a precursor of many bioinspired materials and it is, therefore, a good candidate to investigate on the interplay between dispersion interactions, essentially due to aromaticity, and strong (OH–O) or weak (OH–π) HBs, settled by the hydroxyl substituents. The employed MP2^mod^ computational route consists in MP2 calculations with a small 6-31G** basis set, in which the exponents of the polarization functions are suitably modified. This has been done through a validation procedure based on the comparison with the highly accurate CCSD(T)/CBS calculations, resulting in new exponents for polarization functions on carbon (0.27), hydrogen (0.36), and oxygen (0.34). 

Within the IPES sections explored, two minima were identified, held together by a network of stacking, OH–O, and OH–π interactions, whose relative weight has been analyzed in some detail. The two catechol units tend to aggregate in stacked conformation, which eventually result more stable than the TS ones, thanks to their ability to establish strong and weak HBs.

A final remark should be made concerning the effects that solvation can have in these systems. Despite most computational approaches designed for noncovalent interactions only focus on two isolated molecules, we are aware that water might affect the results and change the picture that we report here (see, for instance, [27,68]). It is, however, important to have a preliminary reference to guide the more complex study in solution, which is a natural continuation of the one presented here. 

## Figures and Tables

**Table 1:** Interaction energies, in kcal/mol, for the four optimized conformations shown in Figure 7, computed with MP2^mod^, CCSD(T)/CBS and MPW1B95/6-311++G(2*d*,2*p*). Central processing unit (CPU) times on a single 2.60 GHz Intel^®^ Xeon CPU are also given for an evaluation of the computational cost of the different methods.

Geometry	Energies (kcal/mol)			CPU Time (min)		
	MP2^mod^	CCSD(T)/CBS	MPW1B95	MP2^mod^	CCSD(T)/CBS	MPW1B95
I	−10.7	−11.1	−8.1	27	50,640	145
II	−12.4	−12.6	−8.3	25	49,740	180
III	−5.3	−5.7	−2.2	27	50,820	79
IV	−6.1	−7.3	−5.7	18	51,720	142
